# Alcohol use, abuse and dependence in an older European population: Results from the MentDis_ICF65+ study

**DOI:** 10.1371/journal.pone.0196574

**Published:** 2018-04-30

**Authors:** Manuel Muñoz, Berta Ausín, Ana B. Santos-Olmo, Martin Härter, Jana Volkert, Holger Schulz, Susanne Sehner, Maria Christina Dehoust, Anna Suling, Karl Wegscheider, Alessandra Canuto, Mike J. Crawford, Luigi Grassi, Chiara Da Ronch, Yael Hershkovitz, Alan Quirk, Ora Rotenstein, Arieh Y. Shalev, Jens Strehle, Kerstin Weber, Hans-Ulrich Wittchen, Sylke Andreas

**Affiliations:** 1 School of Psychology, Complutense University of Madrid, Madrid, Spain; 2 Department of Medical Psychology, University Medical Center Hamburg-Eppendorf, Hamburg, Germany; 3 Department of Psychosocial Prevention, University of Heidelberg, Heidelberg, Germany; 4 Institute of Medical Biometry and Epidemiology, University Medical Center Hamburg-Eppendorf, Hamburg, Germany; 5 Nant Foundation, East Vaud Psychiatric Institute, Geneva, Switzerland; 6 Royal College of Psychiatrist, London, United Kingdom; 7 Institute of Psychiatry, Dpt. Biomedical and Specialty Surgical Sciences, University of Ferrara, Ferrara, Italy; 8 Department of Psychiatry, Hadassah University Medical Center, Jerusalem, Israel; 9 Department of Psychiatry, NY Langone Medical Center, New York, United States of America; 10 Institute of Clinical Psychology and Psychotherapy, Technische Universitaet Dresden, Dresden, Germany; 11 Curabilis, Medical Direction, University Hospitals of Geneva, Geneva, Switzerland; Stellenbosch University, SOUTH AFRICA

## Abstract

**Background:**

Alcohol use disorders (AUD) in older people have been the subject of increasing interest in Europe and worldwide. However, thus far, no reliable data exist regarding the prevalence of AUD in people over the age of 65 years in Europe.

**Objective:**

To assess the current (past month), 12-month and lifetime prevalence of alcohol use, abuse and dependence in people aged 65–84 years.

**Study design:**

The MentDis_ICF65+ study was a representative stepwise cross-sectional survey that was conducted in six European and associated cities (Hamburg, Germany; Ferrara, Italy; London/Canterbury, England; Madrid, Spain; Geneva, Switzerland and Jerusalem, Israel).

**Method:**

In total, 3,142 community-dwelling people aged between 65 and 84 years who lived in participating cities were assessed with an age-sensitive diagnostic interview (CIDI65+).

**Results:**

The prevalence of lifetime alcohol use was 81% for the overall sample. The observed AUD (DSM-IV-TR) prevalence was as follows: current, 1.1%; 12-month, 5.3% and lifetime, 8.8%. Alcohol consumption and AUD were more prevalent in males, and a significant interaction between gender and city was observed; greater gender differences in the prevalence of these disorders were observed in Hamburg, London/Canterbury and Geneva in comparison to the other cities. The prevalence of lifetime alcohol consumption and 12-month AUD tended to be lower in older persons.

**Conclusion:**

The results highlight the appropriateness of using age-adjusted diagnostic tools (CIDI65+) to identify alcohol use and AUD in older people. Different alcohol use patterns were observed in males and females. The results seem to indicate the presence of different alcohol use patterns between northern and southern European countries. Specialized services are proposed, including brief and/or more intensive interventions framed intensive and more simple interventions framed in stepped care strategies, to improve the social and health resources available for older people across Europe.

## Introduction

Alcohol use disorders (alcohol abuse and/or dependence, hereafter, AUD) are among the mental disorders with the highest prevalence rates worldwide [1]. The health risks associated with alcohol consumption increase in older people because their physical tolerance for alcohol decreases while the prevalence of some of the main risk factors for AUD (e.g., life stressors, solitude, social exclusion, physical illness and slower metabolism) is increasing [2,3]. Despite these data, few studies of people over 65 years of age have been conducted [3–6]. The available data indicate that approximately 60% of older people in Western countries have consumed alcohol during the past 12 months [3,4,7], but less than 20% report drinking excessively [6,8,9], and lower levels of alcohol consumption have been identified among those over the age of 65 years compared with younger adults. Thus, the research results on the lifetime prevalence of AUD are very heterogeneous. Reported lifetime prevalence rates include the 7.1% reported by Hanson et al. [10], the 8.9% prevalence of “heavy drinkers” identified by DiBari et al. [8] using biological and psychological screening methods, and the 17.8% prevalence of abuse and 12.5% prevalence of dependence reported by Hasin et al. [11]. The differences observed in studies with representative samples in the United States may be particularly relevant: The National Comorbidity Survey Replication (NCS-R) found prevalence rates of 8.4% [9], whereas NESARC reported 16.1% [12]. Regarding the AUD 12-month prevalence, variation was also observed; reported results vary from 0.1% in the ESEMeD studies [6] to 3% in the National Survey on Drug Use and Health in the United States [7] and from 0.7% in the study by Helmchen in Germany [13] to 0.9% in the Finnish population [14] or 1.5% in the NESARC study conducted in the United States [12]. In a recent meta-analysis of data from 25 epidemiological studies of mental health in older people conducted by Volkert and colleagues [5], only 7 studies included AUD information. Major variations in AUD prevalence were observed across these studies, with a standardized mean of 0.96% (95% CI [0.84–1.07]) and a lifetime AUD prevalence of 11.7% (95% CI [11.08–12.34]). It seems, that recent studies indicate potential biases in the estimation of alcohol use by older people measured through usual epidemiological study approaches [15]. Various factors may explain the differences identified in the reported AUD prevalence among older adults [5]. The definitions of ‘drinker’ or ‘heavy drinker’ vary [16], the sampling methods were not adapted to the cultural context of this population, and the instruments employed were designed for the general population and not for the unique characteristics of older people [17].

## Aims of the study

We established the following study objectives: a) to collect descriptive information on the patterns of alcohol use; b) to identify the current, 12-month and lifetime prevalences of abuse, dependence and AUD; and c) to identify major factors associated with alcohol use and AUD in the population aged between 65 and 84 years residing in six cities in European and European-associated countries.

## Materials and methods

The MentDis_ICF65+ study was a stepwise, cross-sectional, multi-center survey conducted in six European and Europe-associated cities (Hamburg, Germany; Ferrara, Italy; London-Canterbury, England; Madrid, Spain; Geneva, Switzerland; and Jerusalem, Israel). This work was supported by a grant from the European Commission (Grant No: 223105) within the 7^th^ Framework Research Program of the EU. A detailed description of the methodology has been provided elsewhere [18].

### Sample

An age- and gender-stratified random sample of 3,142 older men and women (aged 65–84 years) living in selected catchment areas in communities in each participating country was drawn from population registries in Hamburg (N = 510) and Ferrara (N = 524) and from postal addresses obtained from market research companies in Madrid (N = 555), Geneva (N = 520), London/Canterbury (N = 496) and Jerusalem (N = 542). The inclusion criteria were providing informed written consent, living in the predefined catchment area and being between 65 and 84 years old. The exclusion criteria were severe cognitive impairment, as assessed with the MMSE (Mini-Mental State Examination, cut-off score >18 [18] and an insufficient level of the corresponding language. The response rates for each country were 11% in Hamburg, 17% in Madrid, 19% in Ferrara, 21% in London to 26% in Jerusalem and 31% in Geneva. In an analysis of representativeness, we found only significant differences with a small effect size [19] for the sociodemographic characteristics (marital status, immigration status, work status) between the MentDis_ICF65+ study sample and the population of the respective catchment area and the country of each study center; the exception was for marital status in the Hamburg sample, as the MentDis-ICF65+ study had a lower rate of married elderly adults [19]. The study was approved by research ethics committees in all six centers (Germany: Hamburg Ethics Committee of the Medical Association No. 2895; Italy: Ferrara No. 0096637 5/11/2009; Israel: Jerusalem No. 0376-09-HMO; Spain: Madrid No. 22032010; Switzerland: University Hospitals of Geneva ethics committee, Protocol No. 09-121; and UK: National Research Ethics Service No. 10/H0715/21) [18].

### Measures

Computer-assisted face-to-face interviews of household residents were performed by trained investigators between January and October 2011 using an adapted, age-specific version of the Composite International Diagnostic Interview (CIDI65+) [20]. In accordance with previous work [17], the major modifications implemented were shortening the questions by breaking them down into subsets; using commitment and sensitivity modules consisting of visual aids and dimensional scales to give respondents more time to reconsider and remember; implementing optional synonyms for core symptoms; and reducing the skip rules and extensions of dimensional measures [20]. The CIDI65+ includes a sociodemographic chapter and different sections for diagnosing mental health disorders, including a section designed to assess alcohol use and diagnose AUD according to the Diagnostic and Statistical Manual of Mental Disorders (DSM-IV-TR) [21] criteria. Data regarding past 12-month and lifetime alcohol consumption (drinking any alcoholic beverage more than 12 times) and the diagnosis of AUD were collected. We used a variable (index) that combined the number of alcoholic drinks and their alcohol content (1 drink = 0.10 g alcohol) to quantify the amount of alcohol ingested on a standard day as an indicator of alcohol consumption patterns. To assess these data, we included several specific questions on alcohol use in the CIDI65+. The different sections of the CIDI65+, including the Alcohol section, have been shown to have adequate psychometric properties in the population aged over 65 years [20].

### Statistical analyses

Sample characteristics are provided as absolute and relative frequencies and were calculated for the overall sample and the subsample of drinkers. The survey analysis were weighted (by the number of inhabitants) and stratified (two strata each for gender and age group (65–74 and older than 74 years)). Adjusted prevalence rates (current, 12-month and lifetime abuse, dependence and AUD) were estimated as marginal means based on weighted logistic regression models adjusted for age (in 5-year intervals), gender and city. The differences among cities were analyzed including Bonferroni- adjusted comparisons. The 12-month and highest lifetime indices were estimated as marginal means based on linear regression models with the same adjustment variables. Logistic regression models adjusted for age, gender and city were generated to explore the correlations between major sociodemographic and psychopathological factors and alcohol use, abuse and dependence. Two-way interactions (age*gender and city*gender) were added to and retained in all models if they were found to be significant. Odds ratios (ORs) and their corresponding 95% CIs are presented. For cases in which significant interactions were identified, the OR for the interaction term is presented along with plots of the estimated marginal means (adjusted prevalence rates). All analyses were performed using Stata 12.1. [22]

## Results

The mean age of the participants was 73.7 years, and 50.6% of the participants were female. Additional sociodemographic characteristics are shown in Table 1.

**Table 1:** Sociodemographic characteristics of the overall sample and the subsample of lifetime drinkers (people who reported drinking any type of alcoholic beverage at least 12 times during their lifetime).

		Overall sample N = 3,140	Overall sample N = 3,140	Lifetime drinkers N = 2,390	Lifetime drinkers N = 2,390
		n	%	n	%
Gender	Female	1,590	50.6%	1,045	43.7%
	Male	1,550	49.4%	1,345	56.3%
Age (years)	65–69	918	29.2%	746	31.2%
	70–74	797	25.4%	615	25.7%
	75–79	838	26.7%	636	26.6%
	80–84	587	18.7%	393	16.4%
Marital status	Married	1,915	61.1%	1,537	64.4%
	Separated/divorced/widowed	1,080	34.4%	747	31.3%
	Never been married/other	142	4.5%	103	4.3%
Living alone	No	2,183	69.7%	1,720	72.2%
	Yes	948	30.3%	664	27.9%
Financial situation	Very good	356	11.4%	288	12.1%
	Good	1,371	43.8%	1,087	45.7%
	Just enough	1,145	36.6%	854	35.9%
	Poor	218	77%	136	5.7%
	Very poor	37	1.2%	16	0.7%
Religious affiliation	Very important	827	26.5%	562	23.6%
	Somewhat important	957	30.6%	731	30.8%
	Not very important	660	21.1%	533	22.4%
	Not at all	680	21.8%	551	23.2%
Years of schooling (cut-off 13 years):	Mean (SD)	10.3 (3.2)		10.5 (2.9)	

Regarding the alcohol use patterns observed in the overall sample, 67.8% [62.0;73.7] of the sample had consumed alcohol during the past 12 months, and 81.4% [75.3;87.5] had consumed alcohol at least once over the course of their lives. The indices for alcohol intake on a standard day during the past 12 months and over the course of the participants’ lifetime were calculated, and similar values were obtained for both periods (12-Month: 3.0 [2.7;3.4]; lifetime: 3.3 [2.2;4.5]) (see Table 2).

**Table 2:** Past 12-month and lifetime alcohol use patterns (use of alcohol, and consumption indicator: Index*) in the overall sample and the subsample of lifetime drinkers (people who reported drinking any type of alcoholic beverage at least 12 times during their lifetime).

Overall sample N = 3,140 Alcohol use 12-Month	Overall sample N = 3,140 Alcohol use Lifetime	Lifetime drinkers N = 2,390 Index* 12-Month	Lifetime drinkers N = 2,390 Index* Highest Lifetime
Percentage 95% CI	Percentage 95% CI	Mean (Median) 95% CI [IQR]	Mean (Median) 95% CI [IQR]
67.8% 62.0%-73.7%	81.4% 75.3%-87.5%	3.0 (2) 2.7–3.4 [0;4]	3.3 (0) 2.2–4.5 [0;4]

Note:

*12-month index = measured based on the type and quantity of alcoholic drinks ingested on one standard day during the past 12 months; Highest lifetime index = measured based on the type and quantity of alcoholic drinks ingested on one standard day during the period of their life when they drank most frequently; 1 drink = 0.10 g alcohol; CI = confidence interval.

Table 3 presents the following estimated prevalence rates: current, 12-month and lifetime for abuse, dependence and AUD.

**Table 3:** Current (last month), 12-month and lifetime prevalence of alcohol abuse, alcohol dependence and alcohol abuse and/or dependence disorders (AUD) by cities.

	Current %	Current 95% CI	Group*	12-Month %	12-Month 95% CI	Group*	Life-time %	Life-time 95% CI	Group*
**Abuse**			<0.001			<0.001			<0.001
Hamburg	1.6	1.0–2.2	B	7.1	5.0–9.3	B	12.4	9.0–15.8	C
Ferrara	0.2	0.0–0.3	A	0.8	0.5–1.2	A	1.3	0.7–1.9	A
London	1.3	0.8–1.8	B	8.8	5.7–11.9	B	13.0	9.7–16.2	C
Madrid	-	-	-	1.1	0.1–2.2	A	3.4	1.1–5.7	A B
Geneva	4.3	2.8–5.9	C	8.7	4.9–12.6	B	13.6	8.3–18.9	C
Jerusalem	0.5	0.1–0.8	A	1.2	0.2–2.2	A	3.0	2.2–3.9	B
**Overall**	**1.2**	**0.6–1.8**		**5.0**	**2.0–7.9**		**8.3**	**4.0–12.7**	
**Dependence**						0.003			<0.001
Hamburg	n.e	n.e	n.e	1.0	0.6–1.5	A B	2.4	1.3–3.5	A B
Ferrara	n.e	n.e	n.e	0.5	0.1–1.0	A B	0.8	0.3–1.4	A
London	n.e	n.e	n.e	2.8	1.2–4.3	B	3.8	1.9–5.8	B
Madrid	n.e	n.e	n.e	0.7	0.3–1.1	A B	1.9	0.8–3.0	A B
Geneva	n.e	n.e	n.e	2.2	1.1–3.3	B	3.2	1.1–5.3	A B
Jerusalem	n.e	n.e	n.e	0.2	0.0–0.8	A	0.8	0.6–1.1	A
**Overall**				**1.2**	**0.4–2.1**		**2.3**	**1.2–3.4**	
**AUD**			<0.001			<0.001			<0.001
Hamburg	1.8	1.5–2.1	B	7.4	5.5–9.2	B	12.9	9.8–16.0	C
Ferrara	0.2	0.0–0.4	A	1.0	0.5–1.5	A	1.7	0.9–2.4	A
London	1.4	1.0–1.8	B	9.5	6.4–12.6	B	13.8	10.5–17.1	C
Madrid	0.2	0.0–0.5	A	1.3	0.3–2.2	A	3.8	1.5–6.1	A B
Geneva	4.9	3.5–6.3	C	9.1	5.7–12.5	B	14.1	9.5–18.8	C
Jerusalem	0.5	0.1–0.9	A	1.2	0.2–2.2	A	3.2	2.4–3.9	B
**Overall**	**1.1**	**0.5–1.7**		**5.2**	**2.3–8.2**		**8.8**	**4.5–13.2**	

Note:

*cities sharing a letter in the group columns are not significantly different at the Bonferroni adjusted 5%- level.

n.e. = not estimable.

As shown in Table 3, AUD had the highest overall prevalence, at 8.8% [4.5;13.2]; its lifetime prevalence was 5.3% [2.3;8.2], and it had the lowest prevalence during the past year (12-month) and the last month (current) at 1.1% [0.5;1.7]. Similar trends were identified in the prevalence of alcohol abuse (current: 1.2% [0.6;1.8]; 12-month: 5.0% [2.0;7.9]; lifetime: 8.4% [4.0;12.7]) and dependence (current: not estimable; 12-month: 1.2% [0.4;2.1]; lifetime: 2.3% [1.2;3.4]). In relation to the different cities participating in the study, a trend towards higher prevalence rates for all categories (abuse, dependence and AUD) and all reference periods was observed. The prevalence was higher in Hamburg, London-Canterbury and Geneva and lower in Ferrara, Madrid and Jerusalem (lowest in all categories). At the 12-month timepoint, differences of up to 5 times more AUD were observed in Hamburg, London and Geneva compared to Madrid, Ferrara and Jerusalem. The differences followed the same sense in the Abuse category and, although the same pattern was maintained, the differences in the Dependency category were minor. The results showed similar values for the current and lifetime prevalence.

**Fig 1:**
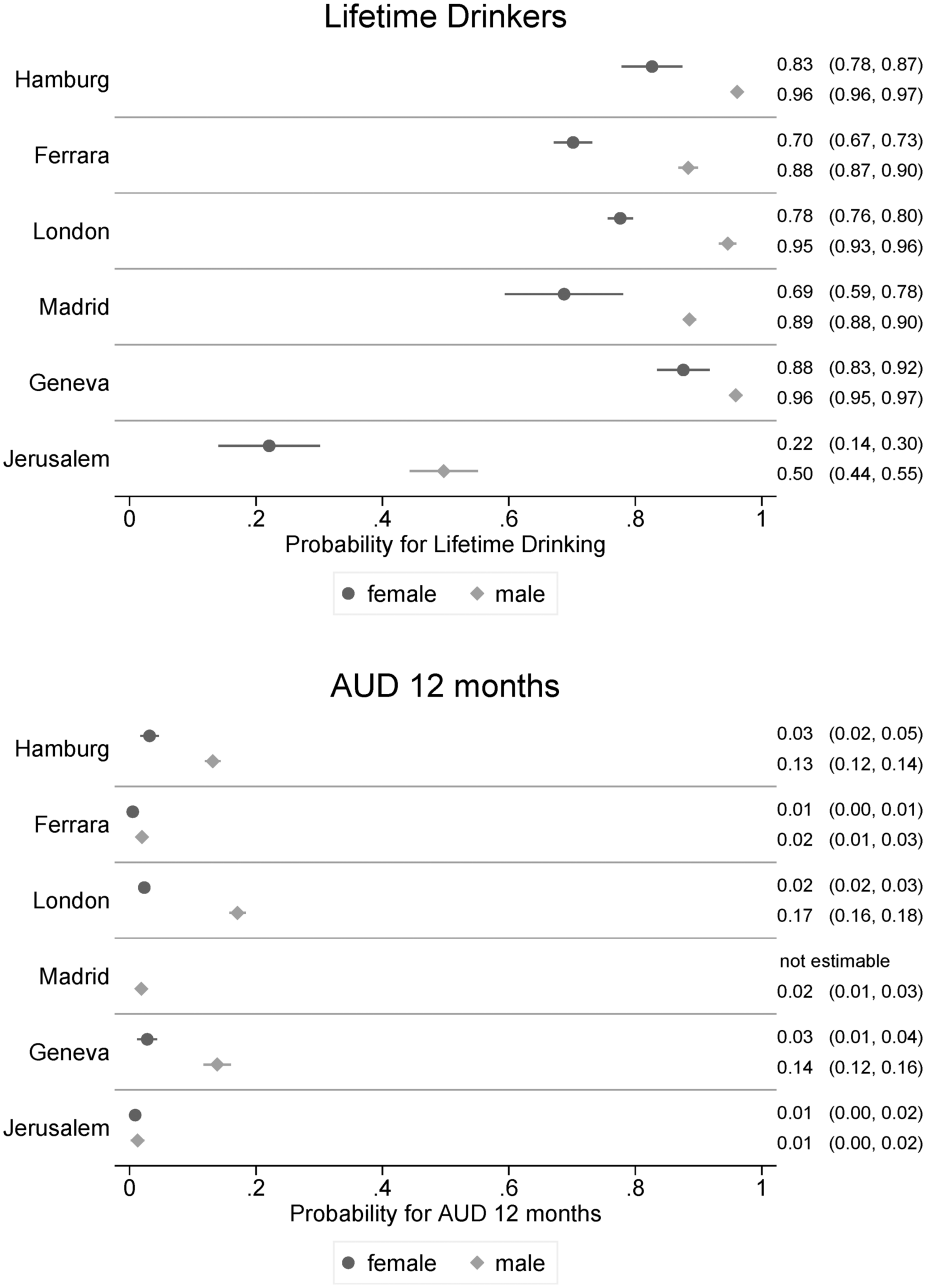
Marginal means (adjusted prevalence) for significant gender*city interactions in lifetime drinkers and people with AUD in the past 12 months. Note: No women in Madrid met the criteria for past 12-month AUD.

The major factors associated with alcohol consumption (lifetime drinkers) and past 12-month and lifetime AUD were identified. Table 4 shows the ORs associated with the main sociodemographic and psychopathological variables, including the city in which the interview was performed. A significant gender*city interaction was identified for lifetime drinking and past 12-month AUD, and Fig 1 displays the adjusted prevalence for both genders within the cities.

**Table 4:** Odds ratios for the associations between alcohol use disorders and sociodemographic characteristics and other mental disorders in the sample of lifetime drinkers and the sample of people who reported alcohol abuse and/or dependence disorders (AUD).

	Lifetime drinkers* OR	Lifetime drinkers* 95% CI	p	12-month* OR	12-month* 95% CI	p	AUD Lifetime OR	AUD Lifetime 95% CI	p
**Gender (male)**	n.e.	n.e.	n.e.	n.e.	n.e.	n.e.	5.3	4.1–6.9	<**0.001**
Madrid	3.8	2.5–5.9	<0.001	n.e.	n.e.	n.e.			
Hamburg	5.5	4.1–7.5	<0.001	5.6	3.6–8.7	<0.001			
Ferrara	3.5	2.9–4.1	<0.001	4.1	1.6–10.2	0.005			
London	5.4	4.1–7.3	<0.001	11.2	8.4–15.0	<0.001			
Geneva	3.4	2.5–4.7	<0.001	6.9	3.7–12.8	<0.001			
Jerusalem	3.8	2.4–5.9	<0.001	1.5	0.4–4.9	0.532			
**Age**			**0.001**			**0.004**			**0.001**
65–69 years (R.G.)									
70–74 years	1.0	0.5–2.0	0.975	0.5	0.4–0.8	0.002	0.7	0.6–0.9	0.011
75–79 years	0.9	0.6–1.3	0.574	0.4	0.3–0.6	<0.001	0.5	0.4–0.7	<0.001
80–84 years	0.6	0.5–0.9	0.022	0.4	0.3–0.7	0.002	0.4	0.3–0.6	<0.001
**Marital status**			0.409			0.317			0.240
Married (R.G.)									
Separated/divorced/widowed	0.7	0.4–1.2	0.181	0.7	0.3–1.7	0.445	0.8	0.5–1.6	0.560
Never been married/other	0.8	0.3–1.8	0.492	0.5	0.2–1.5	0.223	0.5	0.2–1.5	0.199
**Lives alone**									
No (R.G.)									
Yes	1.2	0.7–2.0	0.525	2.0	1.0–3.8	0.042	1.2	0.6–2.4	0.528
**Financial situation**			0.152			**0.016**			**0.005**
Very good (R.G.)									
Good	1.2	0.7–2.0	0.488	0.6	0.3–1.2	0.170	0.7	0.4–1.1	0.093
Just enough	1.3	0.7–2.2	0.355	0.4	0.2–1.3	0.140	0.6	0.4–0.9	0.025
Poor	1.2	0.5–2.6	0.662	2.9	1.1–7.6	0.035	1.8	1.0–3.3	0.065
Very poor	0.6	0.2–2.1	0.363	n.e.	n.e.	n.e.	0.5	0.1–4.0	0.518
**Religious affiliation**			**0.005**			**0.015**			**0.047**
Very important (R.G.)									
Somewhat important	1.4	1.2–1.7	0.001	2.3	1.0–5.2	0.044	0.8	0.6–1.2	0.329
Not very important	1.5	1.1–2.2	0.018	4.7	1.3–16.7	0.019	1.1	0.7–2.0	0.617
Not at all	2.0	1.2–3.3	0.008	3.6	1.8–7.5	0.002	1.3	0.9–2.0	0.160
**Years of schooling**	1.1	1.1–1.2	**<0.001**	1.0	0.9–1.0	0.390	1.0	0.9–1.1	0.815
**City**									**<0.001**
Madrid (R.G.)									
Hamburg	n.e.g.	n.e.g.	n.e.g.	n.e.g.	n.e.g.	n.e.g.	5.4	2.9–10.0	<0.001
Ferrara	n.e.g.	n.e.g.	n.e.g.	n.e.g.	n.e.g.	n.e.g.	0.5	0.3–1.0	0.059
London	n.e.g.	n.e.g.	n.e.g.	n.e.g.	n.e.g.	n.e.g.	5.1	2.5–10.2	<0.001
Geneva	n.e.g.	n.e.g.	n.e.g.	n.e.g.	n.e.g.	n.e.g.	5.4	2.7–10.8	<0.001
Jerusalem	n.e.g.	n.e.g.	n.e.g.	n.e.g.	n.e.g.	n.e.g.	0.9	0.5–1.6	0.694
**Nicotine dependence**	1.4	0.7–3.0	0.341	4.6	2.6–8.0	**<0.001**	3.9	2.5–6.0	**<0.001**
**Any psychotic disorder**	3.5	0.2–72.7	0.395	n.e.	n.e.	n.e.	n.e.	n.e.	n.e.
**Any anxiety disorder**	0.8	0.4–1.3	0.300	1.1	0.6–2.2	0.686	1.4	0.9–2.1	0.115
**Any depressive disorder**	1.8	0.4–8.5	0.429	0.1	0.0–0.3	**0.001**	0.5	0.1–1.4	0.167
**Any affective disorder**	0.5	0.2–1.5	0.186	4.3	1.8–10.1	**0.002**	2.0	0.7–6.1	0.190

Note:

*Significant gender by city interaction,

OR = odds ratio, CI = confidence interval, n.e. = not estimable, n.e.g. = Overall effects not estimable due to significant interaction with gender,

R.G. = Reference group

Regarding lifetime AUD prevalence, males had a 5-times-greater odds of AUD than women (OR = 5.3 [4.1;6.9]). Differences between cities were also identified: residents of Hamburg (OR = 5.4 [2.9;10.0]; p = 0.001), London-Canterbury (OR = 5.1 [2.5;10.2]; p = 0.001) and Geneva (OR = 5.4 [2.7;10.8]; p = 0.001) had a 5-times-greater odds of lifetime AUD than did residents of Madrid, whereas the odds of AUD in Ferrara (OR = 0.5 [0.3;1.0]; p = 0.059) and Jerusalem (OR = 0.9 [0.5;1.6]; p = 0.694) did not differ significantly from those in Madrid. Regarding lifetime drinking and 12-month AUD, we identified a significant gender*city interaction, indicating that the observed gender differences were not the same in all countries. Fig 1 shows that the prevalence of lifetime drinking was the lowest in Jerusalem. Moreover, the odds of lifetime drinking in Madrid, Ferrara, Geneva and Jerusalem were 3–4 times higher in males than females, whereas in London and Hamburg, the odds were 5–6 times higher in males than females. No significant gender difference was identified in the prevalence of 12-month AUD in Jerusalem (prevalence in both genders: 0.01, with an OR of 1.5 [0.4;4.9] for males; p = 0.532), whereas in all other cities, significant gender differences were observed, with the greatest difference identified in London (OR = 11.2 [8.4;15.0]) and the smallest difference identified in Ferrara (OR = 4.1 [1.6;10.2]). No women in Madrid met the criteria for past 12-month AUD (see Table 4, Fig 1).

The prevalence of lifetime alcohol consumption and 12-month and lifetime AUD tended to be lower in the older age groups. There were no significant differences in terms of marital status. The residence situation (living alone) was significantly associated with 12-month AUD only, and living alone was associated with a 2-fold increase in the odds of AUD (OR = 2.0 [1.0;3.8]). Financial situation was significantly and inversely associated with AUD (past 12-month and lifetime: p = 0.016, p = 0.005). Similarly, religious affiliation demonstrated an inverse association with lifetime drinking and past 12-month and lifetime AUD (p = 0.005; p = 0.015; p = 0.047). The odds of lifetime drinking increased by 10% with every additional year of schooling (OR 1.1 [1.1;1.2]) (see Table 4); however, no significant association was identified between schooling and AUD.

Past 12-month and lifetime AUD were associated with nicotine dependence (OR = 4.6 [2.6;8.0]; p = 0.001 and OR = 3.9 [2.5;6.0]; p = 0.001, respectively). There were also significant associations between having any depressive disorder (Major Depressive Disorder, Dysthymia) (OR = 0.1 [0.0;0.3]; p = 0.001) or any affective disorder (Major Depressive Disorder, Dysthymia, Any Bipolar Disorder) (OR = 4.3 [1.8;10.1]; p = 0.002) in the past year and alcohol abuse or dependence during the past 12 months (past 12-month AUD; see Table 4).

## Discussion

This study found higher alcohol consumption and prevalence rates (current, 12-month and lifetime) of abuse, dependence and AUD than previous studies. Significant differences were also identified by gender and city.

The relative novelty of these results can be interpreted from two complementary viewpoints. On the one hand, the sampling strategy employed herein allowed us to include people over 80 years of age. Additionally, the instrument developed and employed in the study (CIDI65+) has shown good reliability in older people [20] for identifying different patterns of use, abuse, and dependence. We detected rates similar to those of previous studies in terms of lifetime consumption and prevalence using these strategies; however, we also identified noteworthy differences between our findings and the results of prior studies in terms of current (past month) and past 12-month prevalence. The higher prevalence rates identified herein may have occurred because non-age-adjusted instruments might produce biases (i.e., lower estimates) in older people [20,17].

Approximately 80% of the participants reported a lifetime consumption of alcohol, and over 65% reported 12-month consumption [4,7]. The following gender and age differences were identified: consumption was higher in males and lower in the older age groups. The odds of reporting alcohol consumption were almost twice as high in the 65- to 70-year age group than in the 80- to 84-year age group. For the recent timepoints (12 months and 1 month), this finding may be due to illnesses (and medications) that are incompatible with alcohol, greater diet control, slower metabolism, etc. However, for the lifetime prevalence, this phenomenon could be explained better by a cohort effect or some type or recall bias in the older population.

Moreover, many people who abuse alcohol may die before reaching old age (80 to 84 years) due to alcohol-induced illnesses. In contrast with the results of prior studies [7], the odds of consumption were only slightly higher in people with more years of schooling, but this association had no impact on past 12-month or lifetime AUD prevalence. The restricted age range for *years of schooling* variable (cut-off: 13 years) used in the present study may have contributed to the observed differences.

The prevalence of lifetime AUD in the sample is consistent with the results of the NCS-R [9] and DiBari et al. [8] studies, which were conducted using biological and behavioral markers in European samples, but were somewhat below the mean values obtained in the meta-analysis performed by Volkert et al. (11.71%) [5] and much lower than the values reported for the NESARC study [12]. Again, the oldest groups (80–84 years old) presented the lowest AUD prevalence. Moreover, gender differences in lifetime prevalence were found to be relevant. Similar to consumption, alcohol-related deaths, diseases and medications may explain the observed decrease in prevalence rates.

One of our most striking findings was that the 12-month prevalence data indicate relatively high levels of alcohol abuse, levels that were higher than those observed in previous studies. This level of abuse was far higher than the 0.96% reported by Volkert et al. (2013) [5] in their meta-analysis, the 0.1% identified in the ESEMeD [6] studies, the 0.7% identified by Pirkola et al. [14) and the 0.9% observed by Helmchen et al. [13], all of which included European samples. The results also deviate from North American studies, which have indicated prevalence rates ranging from 1.5% in the NESARC study [12] to 2.98% in the NSDUH study [7]. However, the results of this study were consistent with those of Waern et al. [23], who, in a recent cohort study conducted in Sweden, found that alcohol consumption in 75-year-olds had increased from 1975 to 2005 in both genders and all the categories studied (e.g., beer, wine, spirits, total grams of alcohol and times per week). These authors reported that the prevalence rates of at-risk drinking (this rate did not include diagnoses) were 19.3% in men and 0.6% in women for the earlier-born cohort (1901) and 27.4% in men and 10.4% in women for the later-born cohort (1930).

In general, these results were in accordance with previous studies that reported positive associations between AUD and affective and other substance use disorders (nicotine) in general population samples [24, 25]. More recent work has also revealed strong relationships between these factors and alcohol dependence [26, 27].

The differences in consumption and AUD prevalence observed among study cities may be particularly relevant. These differences (more consumption, abuse and AUD in Hamburg, London-Canterbury and Geneva) may be related to cultural patterns of alcohol consumption, as some European studies have suggested. The Food in Later Life Project [28] seems to identify different social patterns of alcohol use in Mediterranean countries versus central European and Nordic countries, and the IAS [29] suggested similar results. From a more international perspective, the WHO [30] has reported the existence of different alcohol consumption patterns in different cultural settings, showing that alcohol consumption may be culturally mediated (e.g., Mediterranean countries with a culture of wine production and consumption vs. central European countries and Great Britain, which have a higher consumption of distilled alcohol or beer). However, our findings should be interpreted with caution as we also observed a strong interaction between city and gender, which precludes simple interpretations of consumption and past 12-month prevalence. Both genders exhibited greater odds of alcohol consumption in Hamburg, London/Canterbury and Geneva than in Madrid. The past 12-month prevalence rates showed similar differences, but they were only significant for males.

To increase life expectancy and improve the health and quality of life of older people, it is essential to attend to their physical and mental health [31]. Despite the significant presence of mental health problems in the elderly, the evidence reveals a dual reality. On the one hand, there is a lack of specialized mental health services and interventions designed for the elderly [32, 33], and on the other hand, elderly adults in some countries have difficulty accessing specialized services [34, 35, 36].

A special point about the use of DSM-IV-TR criteria as a measure of psychological disorders among older adults should be noted. The DSM criteria probably underestimate the prevalence of depression and anxiety among older adults [17, 37]. This point could be related to the risk of having to respond to the difficulty to remember the symptoms or the difficulty to associate the symptoms with the psychological distress, among others [20]. Considering the previous facts and despite the limitations of the study (e.g., a relatively low sample size by city, resulting in a low test power; inclusion of a community-dwelling population only; possible recall bias related to lifetime consumption) the evidence suggests that action should be taken to ensure that older people with mental problems receive the best possible care. In this sense, different complementary pathways of action are proposed:

- Designing specialized health care services and interventions for older people that include comprehensive care strategies to address comorbidities and the specifics of physical and psychological disorders in this population. According to the NICE guidelines, the best treatment options for mental health problems in the elderly combine medical and psychological treatments [38]. Older people show motivation to change (including abstain) and they respond well to brief advice and motivational therapy [33]. Furthermore, recent studies suggest that intensive interventions with personalized feedback, physician advice, educational materials plus follow-up could be effective [39].
- Implementing psychological awareness programs in the primary care setting to identify possible psychological and mental disorders and offer stepped interventions that can be adapted to individual needs [40]. In some cases, simple interventions (e.g., brief interventions combined with advice to reduce drinking) could also have a positive effect [39] and could be used in a stepped intervention model.
- Optimizing this population’s access to existing specialized mental health services in each city.

These efforts may include supporting social transformation processes that strive to eliminate the social exclusion of older people with mental disorders and designing a new generation of social and health services that recognize the specific needs of the older population from a holistic and inclusive perspective.
